# Polymeric Surfactant (PIBSA-X) Facilitates the Formation of a Water-in-Oil Emulsion Reactor for the Preparation of Ultrasmall Nanosilica

**DOI:** 10.1021/acsomega.3c05335

**Published:** 2023-11-14

**Authors:** Rui Cao, Chun Wang, Chengliang Zhou, Yong Liu, Yating Yin, Haibao Chen, Feng Li, Wending Zhou, Meisong Xu, Wanliang Yang

**Affiliations:** † School of Chemistry and Chemical Engineering, Guizhou University, Guiyang 550025, China; ‡ Guizhou Juneng Chemical Co, Ltd, Huishui County of Guizhou Province, Huishui 550601, PR China; § Guizhou Provincial Double Carbon and Renewable Energy Technology Innovation Research Institute, Guizhou University, Guiyang 550025, China

## Abstract

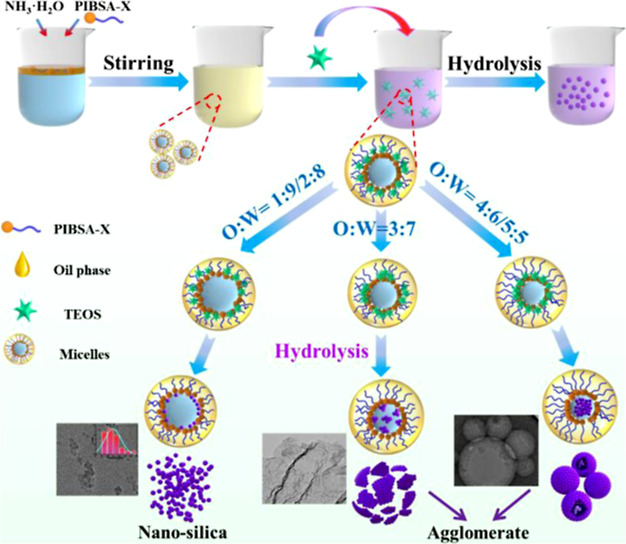

Despite the widespread application of ultrasmall nanosilica, solving its aggregation problem during the preparation process remains a challenge. In this paper, ultrasmall nanosilica with a controllable size and aggregates were prepared through the water-in-oil (W/O) emulsion method by using polyisobutylene succinic anhydride-type polymeric surfactants (PIBSA-X) as an isolating agent. PIBSA-X polymeric surfactants with different hydrophilic groups were prepared using industrial-grade PIBSA, which can form stable W/O-type emulsions well. Subsequently, the W/O-type emulsion droplets were used as reactors and tetraethyl orthosilicate was hydrolyzed under ammonia alkaline conditions to synthesize ultrasmall nanosilica (10 nm). Furthermore, the morphological evolution of nanosilica aggregates can be tuned by varying the oil/water ratio, which controls the emulsion droplets. A possible mechanism is proposed to explain why the emulsion method approach affords nanosilica aggregates with various morphologies and pellet size in water-in-oil (W/O-type) emulsion droplets. This study provides a precise and simple synthetic method for the development of ultrasmall nanosilica, which has good potential to be industrialized.

## 1 Introduction

Silicon dioxide compounds benefits from low thermal conductivity, low refractive index, high optical transparency, and high specific surface area, so it is widely used in catalysis, microelectronics, optical systems, and biomedical fields.^1−4^ The particle size and morphology of silica particles affect their physical, chemical, and optical properties, so it is very important to control the particle size and morphology of silica particles.^5−7^ For example, ultrasmall nanosilica can be used to prepare deep-ultraviolet (UV) light-transmitting coatings, which effectively reduce the light scattered through the coating and thus significantly increase the light transmission of the coating in the deep-ultraviolet wavelength.^8,9^ Cancer drug carriers prepared from silica sol–gel have better biocompatibility with larger drug loading rate.^10^ Therefore, silica with different particle sizes and morphologies has become a hot topic of research for many researchers.^11,12^

Many research groups have prepared nanosilica by the hydrothermal method, microemulsion method, ion exchange method, precipitation method, electrospinning method, and chemical vapor deposition method.^13−17^ Since Stöber, Fink, and Bohn’s pioneering research in 1968 to prepare micrometer-sized spherical silica in the absence of surfactants, Stöber has prepared silica nanoparticles from 50 nm to 1 μm over a long period of research and exploration.^18,19^ However, in order to obtain solid ultrasmall nanosilica, it is necessary to use toxic solvents (methanol)^20^ or to control the synthesis by adding surfactant Triton X-100 as a protectant or amino acids as a catalyst, which results in solid ultrasmall nanosilica that are not useable for practical applications and that are environmentally and biologically hazardous. At the same time, this will significantly increase the cost of production.^9,21^ Commercially available solid nanosilica produced industrially by precipitation is heterogeneous in size, irregular in morphology and difficult to control in the preparation procedure.^22,23^ In comparison with other methods, the ion exchange method can prepare only silica sol–gel. As an industrial production method for commercially available silica sol–gel, the ion exchange method utilizes water glass as the raw material and prepares silica sol–gel^24^ through the process of ion exchange reaction, preparation of crystal seeds, particle growth reaction, concentration step, and purification step, which is characterized by high silica content, uniform particles, and low sodium ion residue. However, each step has a direct effect on the product quality of the final silica sol–gel.

Compared to various preparation methods, the emulsion method has relatively low technical difficulty and is a simple and easy way to prepare nanoparticles.^25,26^ The solid nanosilica prepared by the emulsion method^27^ is uniform in size and completely spherical, and the emulsion method provides a large degree of control over the particle size and morphology of the synthesized ultrasmall nanosilica.^28,29^ The formation of ultrasmall nanosilica is accomplished in water-in-oil (W/O) emulsion droplets-“reactors”, which are used for confined nucleation and growth of ultrasmall nanosilica.^30^ Stabilized water-in-oil antimicellular emulsion droplets are repeatedly moved to prevent excessive aggregation of the generated ultrasmall nanosilica.^31,32^ Because of the thermodynamic instability of emulsions and the large droplet size, the design of the surfactant to the water- and oil-phase interfaces in emulsion polymerization is critical for controlling droplet size, morphology, and stability.^28,33^

Surfactants play a crucial role in the preparation of silica by the emulsion method. In the preparation of ultrasmall nanosilica, surfactants are mainly used as templates or pore-forming guides. Yang’s group successfully synthesized one-dimensional (1D) hollow silica nanospheres (HSNS) with ultrasmall (20 nm) particle size distribution, layered hollow silica spheres (HHSS) composed of self-assembled hollow silica nanospheres, and layered silica (2D) with multiple morphologies by using CTAB as a template.^7,34−36^ Some researchers have also used macromolecular surfactants for the preparation of porous silica, such as the synthesis of highly ordered hexagonal mesoporous silica structures (SBA-15) by Zhao’s group in the presence of large molecular weight triblock poly(ethylene oxide)-poly(propylene oxide)-poly(ethylene oxide) (PEO-PPO-PEO) copolymers.^37^ Huo et al. used nonionic block copolymer (Pluronic F127) micelles as a template to deposit silica in the hydrophilic PEO shell region to form silica nanoparticles of about 12 nm.^38^ Nevertheless, surfactants are used as templates to prepare silica in the emulsion method, and the aggregation of templates often leads to the aggregation of silica. Therefore, the synthesis of nanosilica materials with ultrasmall particle size by the emulsion method is of great scientific interest and remains a great challenge.

In order to solve the aggregation of silica, PIBSA-X, as a long chain surfactant, was designed for preparing ultrasmall (10 nm) silica nanoparticles by forming water-in-oil (W/O) emulsion droplets as a reaction vessel, in which the long hydrophobic chain can isolate the silicon source from approaching. First, we choose industrial PIBSA for a one-step reaction with alcohols, amines, and alcohol-amine reactants with different molecular weights of hydrophilic groups to prepare low-cost, environmentally friendly, and industrialized polymeric surfactant PIBSA-X. Then, the polymeric surfactant PIBSA-X was used as an isolating agent in water-in-oil (W/O) emulsion droplets to obtain ultrasmall silica nanospheres by tetraethyl orthosilicate (TEOS) hydrolysis condensation under alkaline conditions. We also focused on the effects of different hydrophilic groups of polymeric surfactants, oil/water ratio, TEOS addition, and ammonia addition on the morphology and particle size control of solid nanosilica. Finally, we determined the synthesis of polymeric surfactants and the particle size and morphology of solid ultrasmall nanosilica by scanning electron microscopy (SEM), transmission electron microscopy (TEM), TG, and Fourier transform infrared (FT-IR) characterization. To the best of our knowledge, it is the first time an ultrasmall nanosilica system has been designed by using PIBSA-X surfactants.

## 2 Experimental Section

### 2.1 Materials

PIBSA was purchased from Henan Enhydride Co. diethylenetriamine (DETA), triethylenetetramine (TETA), urea, ethylene glycol (EG), diethylene glycol (DEG), triethylene glycol (TEG), *N*,*N*-dimethylethanol, 2-methylaminoethanol, triethanolamine (TEA), and ethyl orthosilicate (TEOS) were obtained from Macklin, China. Ammonia solution (25%) was received from Tianjin Fuyu Fine Chemistry Co., Ltd. Base oil and distilled water were obtained from Guizhou Juneng Chemical Co., Ltd. (Huishui, China).

### 2.2 Preparation of Polymeric Surfactants with Different Hydrophilic Groups

Without solvent, *n* (PIBSA)/*n* (raw material, *X =* 1:2). PIBSA was preheated to 160 °C, waited for PIBSA to be fluid, passed nitrogen to exclude air in the reactor, added raw material (*X*) slowly during stirring, controlled the air bubbles in the reactor when adding *X* to prevent overflow, and continued the reaction for 6 h after dropwise addition is completed. The corresponding reactions were carried out using raw materials with different hydrophilic groups (refer to the reaction steps in the Supporting Information).

### 2.3 Preparation of Ultrasmall Nanosilica

Take 1 g of polymeric surfactant in a beaker, add 20 g of base oil, and mix well under a water bath at 70 °C. Add 80 g of deionized water containing ammonia and stir completely under mechanical stirring for 1 h (at 25 °C). Then, 7.2 mL of TEOS was slowly added (drop by drop, within 5 min), and the reaction was carried out for 3.5 h. The resulting suspension was poured into a Teflon reactor for the hydrothermal synthesis at 100 °C for 24 h.

After crystallization, 5 mL of the crystallized solution was taken into a 50 mL centrifuge tube, 10 mL of ethyl acetate and 10 mL of anhydrous ethanol were added, and the mixture was sonicated for 15 min with a rubber-tipped dropper. After sonication, the solution was centrifuged (10,000 rpm, 3 min) and dried at 80 °C. After drying, calcine was added at 550 °C (5 °C/min) for 6 h.

### 2.4 Emulsification Performance Test Method

0.25 g of polymer surfactant is taken in the reactor, 5 g of base oil is added, and the mixture is mixed thoroughly under high-energy emulsification equipment (as shown in Figure S1, 400 rpm). Then, 20 g of deionized water was slowly added and stirred thoroughly (1200 rpm). Transfer the emulsified liquid to the mill-mouth colorimetric tube and observe whether water and oil precipitate.

### 2.5 Characterization

SEM images were recorded with a Hitachi S-4700 electron microscope. The X-ray diffraction (XRD) patterns were collected by a Bruker D2 ADVANCE instrument, using Cu Kα radiation from 5 to 90° (2θ) with 15°/min. Scanning TEM (STEM) was obtained using a JEM-3010 instrument (JEOL, Japan). Thermogravimetric analysis (TGA) images were obtained by using a STA449C (NETZSCH, Germany) in nitrogen (26–800 °C). The N_2_ adsorption–desorption isotherm was characterized by ASAP2460 (Micromeritics, USA) and the aggregation morphology of emulsion was observed by the polarization microscope (BM2100POL, China).

## 3 Results and Discussion

### 3.1 Characterization and Emulsification Stability of PIBSA-X Industrial-Grade Surfactant

**Figure 1:**
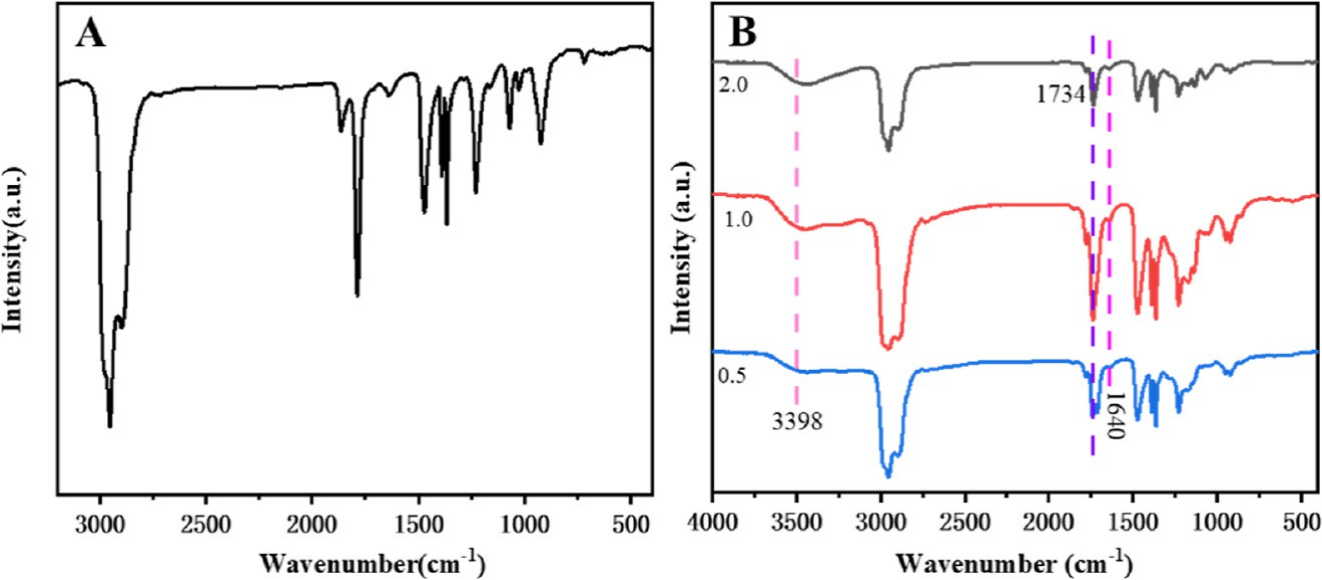
FT-IR spectra of industrial grade PIBSA (A) and PIBSA-TEG polymeric surfactant (B) with different reaction ratios (PIBSA/TEG = 0.5, 1.0, and 2.0).

The polyisobutylene succinic anhydride (PIBSA) used in this experiment is the industrial-grade polyisobutylene succinic anhydride produced by Henan Enhydride Co. The FT-IR spectra are shown in Figure 1A, and the characteristic peaks are shown in Table 1. Industrial-grade polyisobutylene succinic anhydride raw material was selected for the synthesis of polymeric surfactants. ^1^H NMR analysis (Figure S2) of the obtained product with PIBSA revealed that the product could not be characterized due to the presence of impurities; therefore, the presence of the target product was illustrated by comparison of the infrared characterization of the product and PIBSA.

**Table 1:** Peak Attribution of Isobutylene Succinic Anhydride in the Infrared Spectrum

absorption peak (cm^–1^)	attribution peak
2954	–CH_3_, –CH_2_– and C–H str Stöber hing vibrational peaks
1860	pentameric cyclic anhydride asymmetric C=O stretching vibration peak
1787	pentameric cyclic anhydride symmetric C=O stretching vibration peak
1470	–CH_3_, –CH_2–_ and C–H deformation vibration peak
1389	–(CH_3_)_2_ and *tert*-butyl C–H shear vibration peaks
1365	–(CH_3_)_2_ and *tert*-butyl shear vibration peaks
1229	the *tert*-butyl C–C skeleton vibrational peak and the acid anhydride C–O–C vibrational peak overlap
1074	acid anhydride C–O–C vibration peak
921	C–C skeletal vibrational peaks of –(CH_3_)_2_

FT-IR spectroscopy was used to compare the functional groups of PIBSA and PIBSA-TEG. By comparison (Figure 1A) with (Figure 1B), it can be found that the ester carbonyl group appeared as a strong absorption band at 1734 cm^–1^ and the broad band at 3438 cm^–1^ due to terminal (–OH) of ethylene glycol. A comparison of the IR spectra of PIBSA-DEG and PIBSA-EG polymeric surfactants (Figures S3 and S5) with those of PIBSA shows that the C=O stretching vibration peak of the ester group at 1734 cm^–1^ and the alcohol hydroxyl peak at around 3490 cm^–1^ appear in the spectra of both products, indicating that the target products have been synthesized.

**Figure 2:**
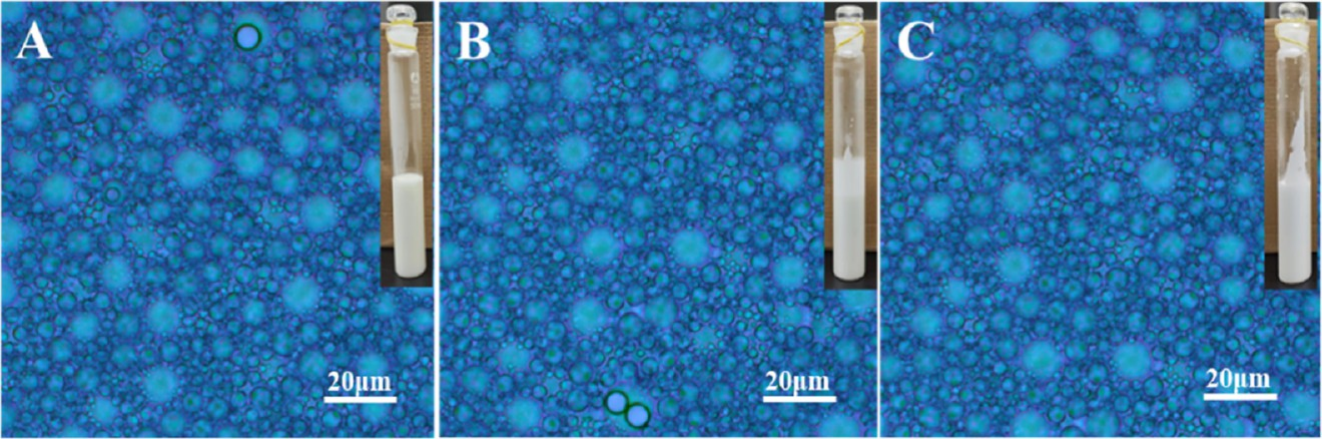
Polarized micrographs and photomicrographs of emulsions emulsified with different reaction ratios of PIBSA-TEG polymer surfactants: (A) 0.5, (B) 1.0, and (C) 2.0.

As shown in Figure 2A–C, it was observed that all reaction ratios (0.5, 1.0, and 2.0) of PIBSA-TEG polymeric surfactant maintained good emulsification even after the 28th day of emulsification under the oil/water ratio of 2:8. From their polarized micrographs, it can be found that the size of emulsified droplets of three samples is at the micrometer level, and all of them are well dispersed. However, PIBSA-DEG polymeric surfactant (Figure S4) and PIBSA-EG polymeric surfactant (Figure S6) did not emulsify as well as PIBSA-TEG polymeric surfactant. This is due to the longer carbon chains of the PIBSA-TEG polymeric surfactants, which resulted in lower interfacial tension and better emulsification.

**Figure 3:**
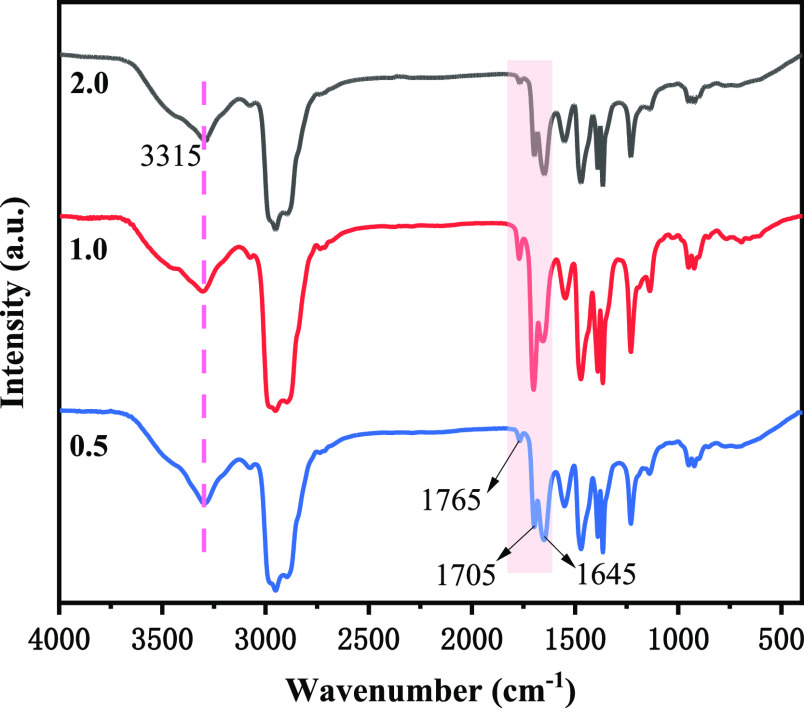
FT-IR spectra of the PIBSA-DETA polymeric surfactant with different reaction ratios (PIBSA/DETA = 0.5, 1.0, and 2.0).

Comparing the FT-IR spectral analysis of PIBSA-DETA polymeric surfactant (Figure 3) with that of PIBSA (Figure 1A). It was found that the imide carbonyl C=O asymmetric stretching vibration peak, carbonyl C=O symmetric stretching vibration peak, and open chain secondary amide carbonyl C=O stretching vibration peak are present in the range of 1645–1800 cm^–1^ for the PIBSA-DETA polymeric surfactant. The anhydride absorption peak at 1855 cm^–1^ on the product spectrum has completely disappeared, so it can be judged that the PIBSA-DETA product has been formed. As shown in the FT-IR spectra of PIBSA-TETA polymer surfactant and PIBSA-urea polymer surfactant (Figures S7 and S9), the imide carbonyl C=O asymmetric stretching vibration peak, the carbonyl C=O symmetric stretching vibration peak, and the open-chain acetamide carbonyl C=O stretching vibration peak were found in the range of 1645–1800 cm^–1^. Such a result shows that the formation of the PIBSA-TETA polymer surfactant and PIBSA-urea polymer surfactant could be determined.^39,40^

**Figure 4:**
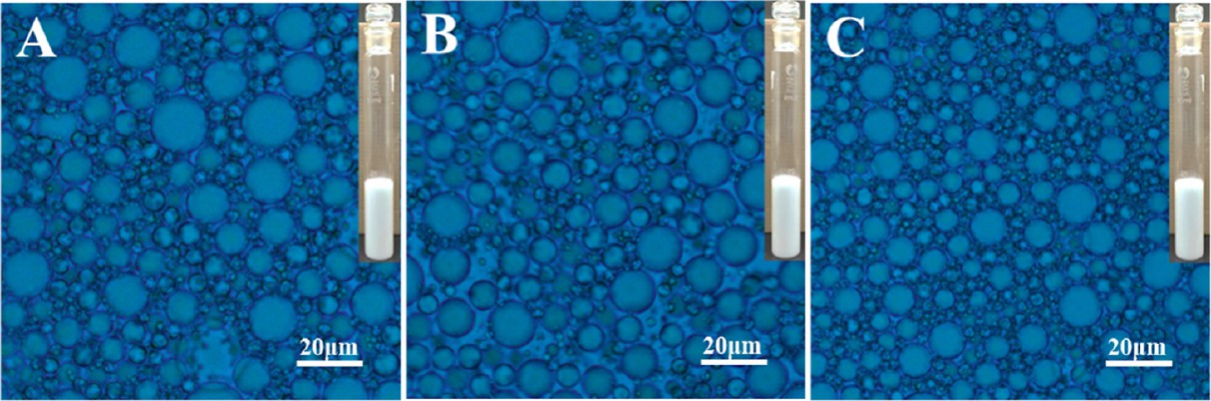
Polarized micrographs and photomicrographs of emulsions emulsified with different reaction ratios of PIBSA-DETA polymer surfactants: (A) 0.5, (B) 1.0, and (C) 2.0.

As shown in Figure 4A–C, this result is for a sample after 28 days of emulsification at an oil/water ratio of 2:8, and it was observed that all reaction ratios (0.5, 1.0, and 2.0) of PIBSA-DETA polymeric surfactant emulsified well. The polarized micrographs of all reaction ratios of PIBSA-DETA polymeric surfactants after emulsification showed that the oil droplet particle size was much smaller than the water droplet and wrapped around the water droplet. It is also found that the 2.0 reaction ratio of PIBSA-DETA polymeric surfactant is easier to obtain a uniform water-in-oil structure after emulsification. Similarly, both the PIBSA-TETA polymeric surfactant (Figure S8) and the PIBSA-urea polymeric surfactant (Figure S10) also have good emulsification performance.

**Figure 5:**
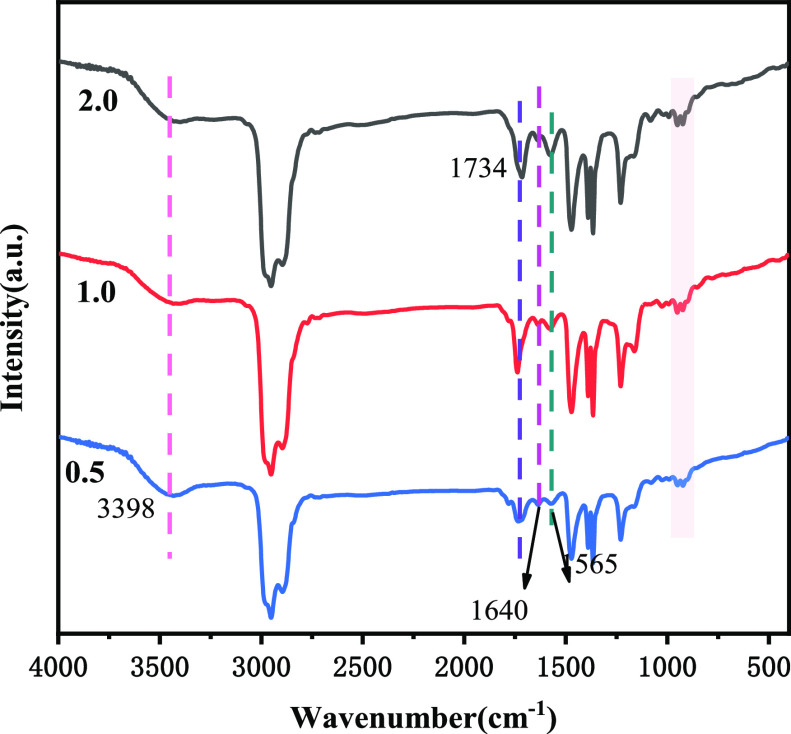
FT-IR spectra of PIBSA-(*N*,*N*-dimethylethanolamine) polymeric surfactant with different reaction ratios [PIBSA/(*N*,*N*-dimethylethanolamine) = 0.5, 1.0, and 2.0].

**Figure 6:**
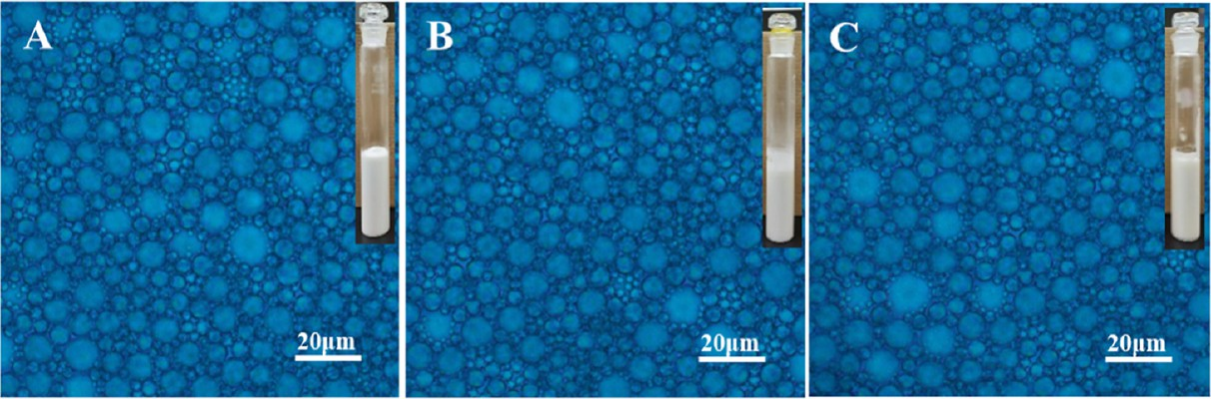
Polarized micrographs and photomicrographs of emulsions emulsified with different reaction ratios of PIBSA-(*N*,*N*-dimethylethanolamine) polymer surfactants: (A) 0.5, (B) 1.0, and (C) 2.0.

The FT-IR diagram of PIBSA-(*N*,*N*-dimethylethanolamine) polymer surfactant (Figure 5) identified an ester bond peak at 1734 cm^–1^ and a hydroxyl peak at 3330 cm^–1^, and a carboxylic acid peak at 1565 cm^–1^. Compared with the FT-IR diagram of PIBSA (Figure 1A), the characteristic peaks of pentacyclic anhydride at 1860 and 1780 cm^–1^ were weakened, which indicated that the anhydride was completely ring-opened during the reaction process and proved that the PIBSA-(*N*,*N*-dimethylethanolamine) polymeric surfactant had been synthesized. Similarly, in the comparison of the IR spectra of PIBSA-(2-methylaminoethanol) and PIBSA-TEA polymeric surfactants with PIBSA (Figures S11 and S13), an ester bond peak around 1730 cm^–1^ and a hydroxyl peak at 3390 cm^–1^ as well as a carboxylate peak at 1570 cm^–1^ are present in the spectra of both products, indicating that the target product has been synthesized. All reaction ratios (0.5, 1.0, and 2.0) of PIBSA-(*N*,*N*-dimethylethanolamine) polymeric surfactant were emulsified at an oil/water ratio of 2:8 and well emulsification was observed on the 28th day of emulsification, as shown in Figure 6A–C. Polarized light micrographs showed that the emulsification of all reaction ratios yielded uniformly dispersed water-in-oil structures. However, the 0.5 reaction ratio of PIBSA-(2-methylaminoethanol) polymeric surfactant (Figure S12A) showed delamination after 20 days of emulsification, indicating that the emulsification of PIBSA-(2-methylaminoethanol) polymeric surfactant was poorly stable. Whereas the 1.0 and 2.0 reaction ratios of PIBSA-(2-methylaminoethanol) polymeric surfactant (Figure S12B,C) and PIBSA-TEA polymeric surface (Figure S14) activator had stable emulsification. ^1^H NMR analysis of the industrial grade PIBSA revealed (Figure S2) that the product obtained was impure due to the choice of industrial grade PIBSA. Therefore, PIBSA-X products could not be characterized by ^1^H NMR analysis.

### 3.2 Characterization of Ultrasmall Nanosilica

#### 3.2.1 Effect of Different Hydrophilic Groups as Isolating Agents for the Preparation of Silica Nanoparticles

**Figure 7:**
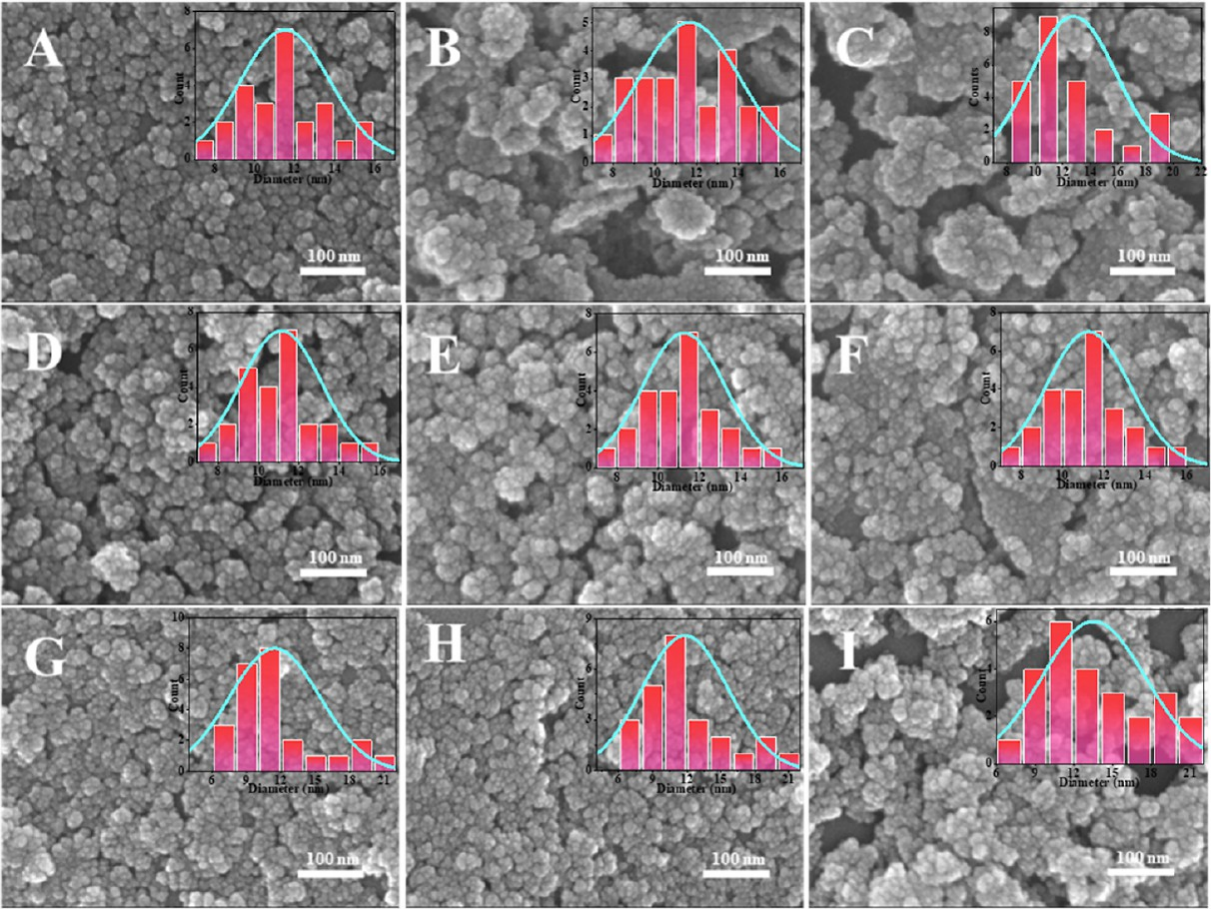
EM image of silica prepared by PIBSA-polyol as an isolating agent: (A–C) PIBSA-TEG (2.0, 1.0, and 0.5), (D–F) PIBSA-DEG (2.0, 1.0, and 0.5), and (G–I) PIBSA-EG (2.0,1.0, and 0.5).

**Figure 8:**
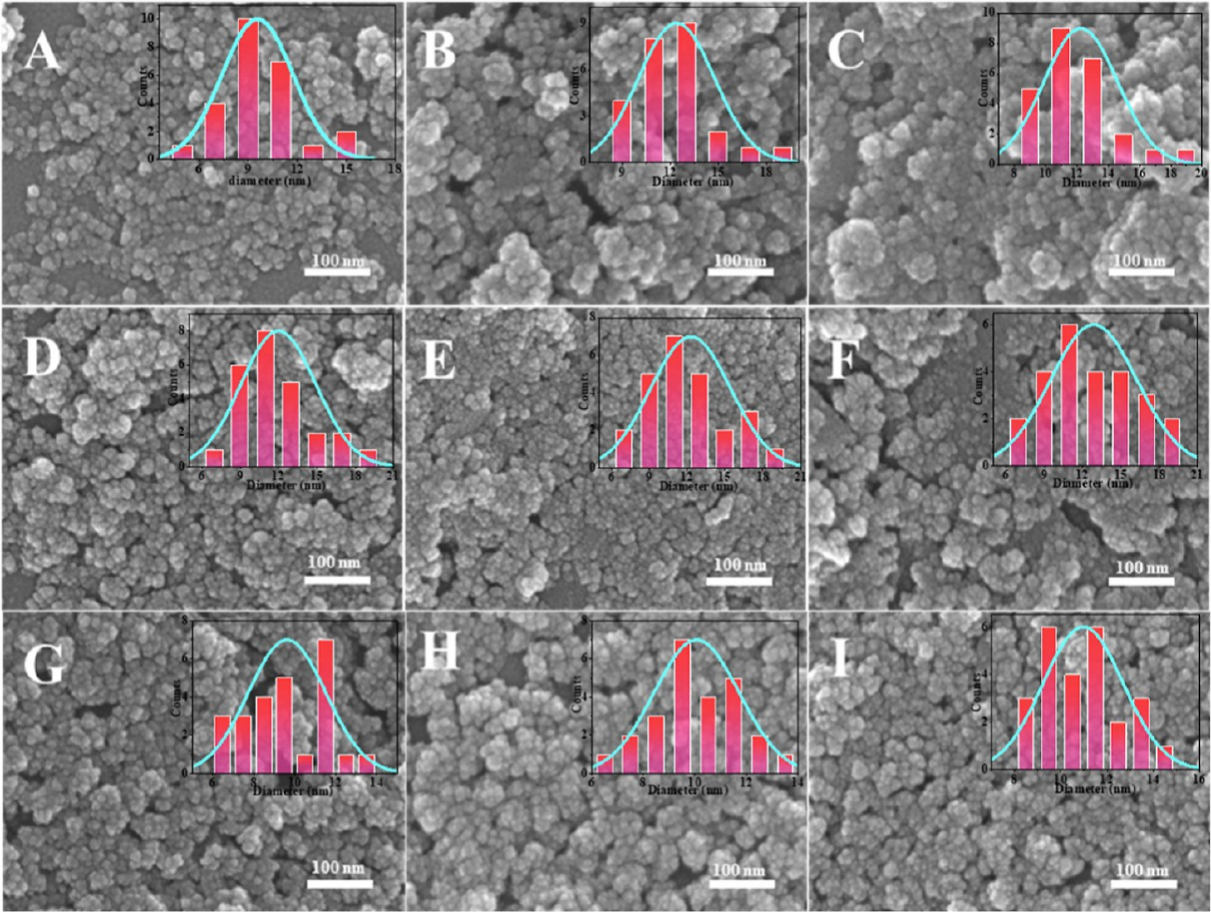
SEM image of silica prepared by PIBSA-polyenamine as the isolating agent: (A–C) PIBSA-DETA (2.0, 1.0, and 0.5), (D–F) PIBSA-TETA (2.0, 1.0, and 0.5), and (G–I) PIBSA-urea (2.0, 1.0, and 0.5).

**Figure 9:**
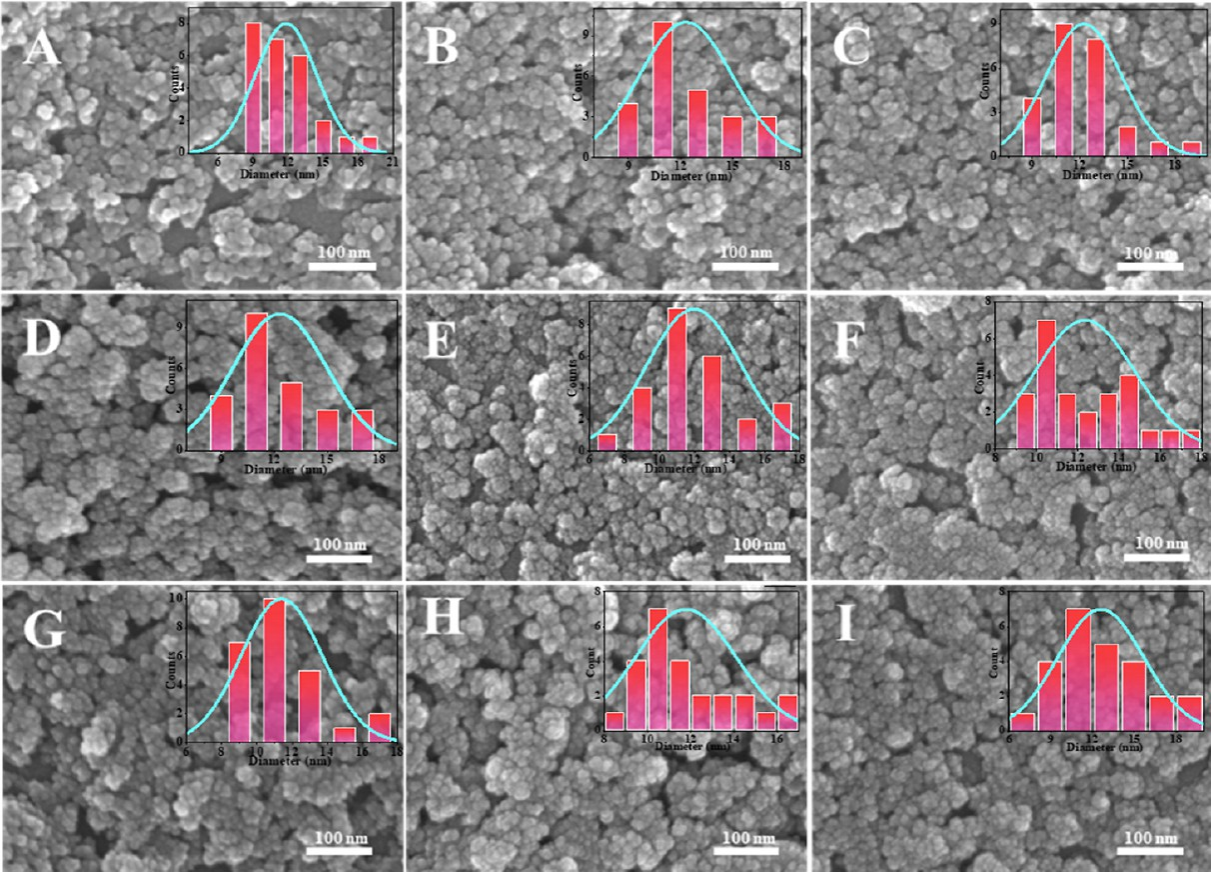
SEM image of silica prepared by PIBSA-polyolamine as the isolating agent: (A–C) PIBSA-*N*,*N*-dimethylethanolamine (2.0, 1.0, and 0.5), (D–F) PIBSA-2-methylaminoethanol (2.0, 1.0, and 0.5), and (G–I) PIBSA-TEA (2.0, 1.0, and 0.5).

The silica was prepared at an oil/water ratio of 1:9, TEOS concentration of 0.072 mol/L, and ammonia concentration of 0.04 mol/L. PIBSA-polyol polymeric surfactant was used as an isolating agent to prepare silica first. Figure 7 shows that different reaction ratios of PIBSA-polyol as isolating agents can prepare silica with a particle size of about 10 nm. 2.0 reaction ratios of alcohol ester polymer surfactants as isolating agents can obtain more dispersed silica. Figures 8A–I and 9A–I show the SEM images of ultrasmall nanosilica nanospheres using different polymeric surfactants, which indicates 2.0 reaction ratios of PIBSA-polyalkylamine and PIBSA-polymer surfactants also can be used to prepare 10 nm and dispersed ultrasmall nanosilica.

**Figure 10:**
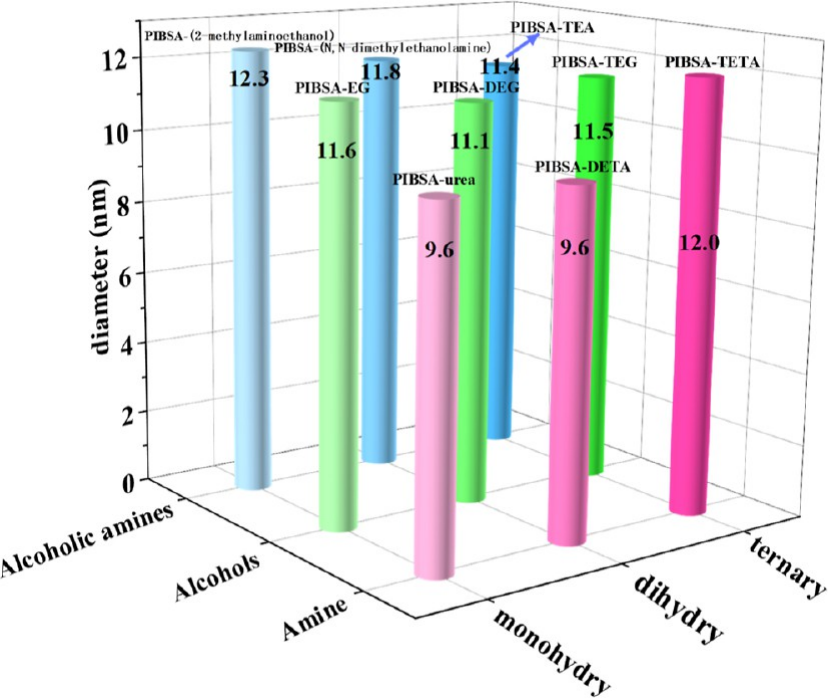
Average particle size of silica prepared by polymeric surfactants with different hydrophilic groups.

Combining the results of these three different hydrophilic group polymeric surfactants that were used as the isolating agent in preparing silica, it is clear that PIBSA-X polymeric surfactant is universal for the preparation of 10 nm silica. That is because the thousands of molecular weight PIBSA-X polymeric surfactant separate TEOS well, allowing the dispersed silica to be prepared universally, which is why the 2.0 reaction ratio of PIBSA-X prepares the most dispersed silica. As can be seen from Figure 10, the average particle size of the ultrasmall nanosilica prepared by the amine polymer surfactant is smaller than ultrasmall nanosilica prepared by the other two polymer surfactants. Comparing the SEM images of the ultrasmall nanosilica obtained from the preparation of PIBSA-DETA polymeric surfactant (Figure 8A–C) and PIBSA-urea polymeric surfactant (Figure 8G–I), the better dispersion and more uniform particle size distribution of the ultrasmall nanosilica prepared from PIBSA-DETA polymeric surfactant can be found. Based on the above analysis, the ultrasmall nanosilica prepared by PIBSA-DETA shows the best dispersion.

#### 3.2.2 Factors Affecting Silica Morphology Using PIBSA-DETA as an Isolating Agent

**Figure 11:**
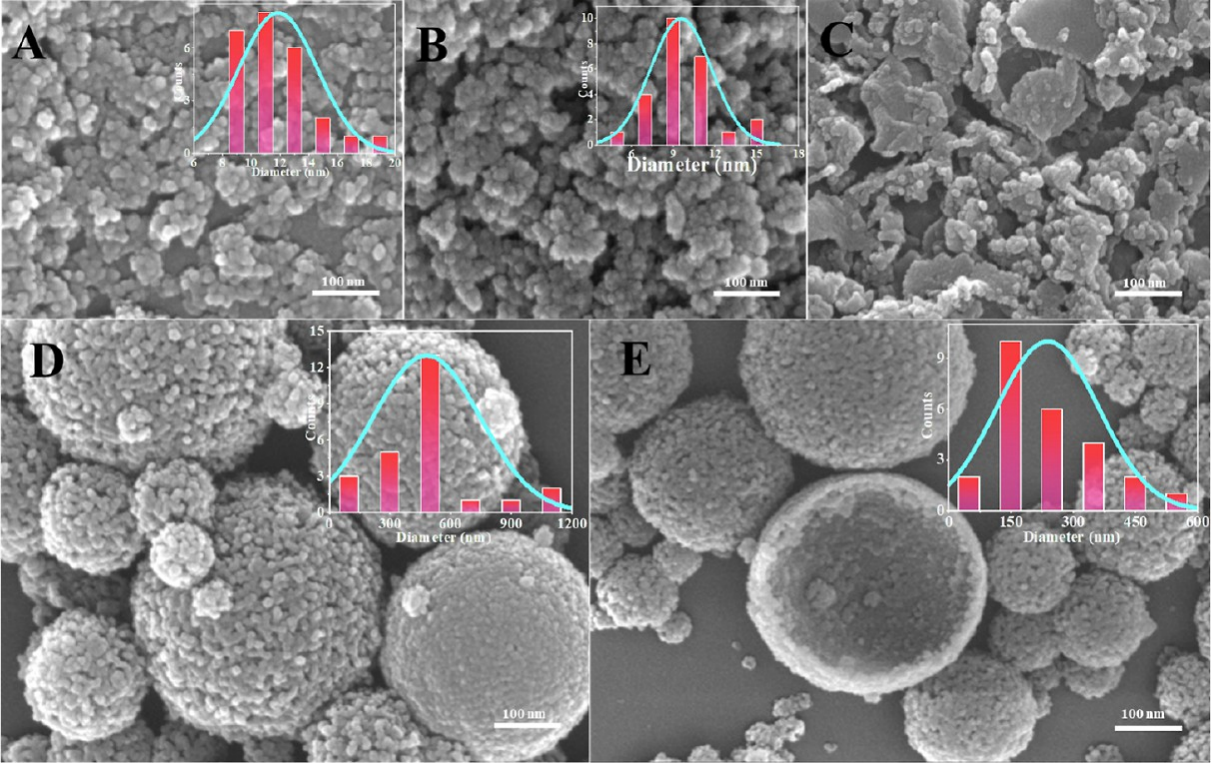
SEM images of silica prepared by different oil/water ratios: (A) 1:9, (B) 2:8, (C) 3:7, (D) 4:6, and (E) 5:5.

To control the morphology of silica nanoparticles, different oil/water ratios were designed for the synthesis of nanosilica by using PIBSA-DETA. The SEM shows that the oil/water ratio increases from 1:9 to 5:5, and the silica goes from dispersed 10 nm spheres to agglomerated into micrometer-sized spheres (Figure 11A–E). At an oil/water ratio of 1:9 and 2:8, the minimum particle size was obtained. This is because TEOS that was isolated the long hydrophobic chain is hydrolyzed at the oil–water interface to generate silica, which enters the water phase through the gap due to the hydrophilic property of silica. When the oil/water ratio becomes larger and the water phase is relatively reduced, resulting in the possibility of collision of silica in the water phase becoming larger, which leads to silica assembly agglomeration into large particles.

**Figure 12:**
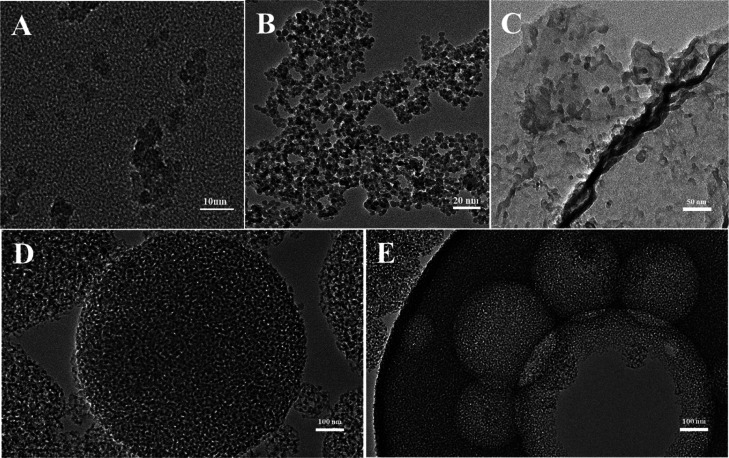
TEM images of silica prepared by different oil/water ratios: (A) 1:9, (B) 2:8, (C) 3:7, (D) 4:6, and (E) 5:5.

TEM images of silica synthesized in a PIBSA-DETA emulsion system with a 2.0 reaction ratio at an ammonia concentration of 0.04 mol/mL and a TEOS concentration of 0.07 mol/mL, with the only change being a change in the oil/water ratio (Figure 12). The oil/water ratio: cyclohexane volume ratio ranges from 1:9 to 5:5. Interestingly, ultrasmall silica nanoparticles with an average size of 10 nm were well dispersed and prepared using PIBSA-DETA polymeric surfactant as the isolating agent and an oil/water ratio of 1:9 and 2:8 (Figure 12A,B). As the oil/water value increases, the water phase decreases, making it easier for silica entering the aqueous phase to aggregate, leading to the formation of lamellar aggregates or even micrometer-sized aggregates by aggregation of 10 nm silica particles (Figure 12C–E). These results are consistent with the SEM images (Figure 11) and our proposed silica formation mechanism (Figure 17).

**Figure 13:**
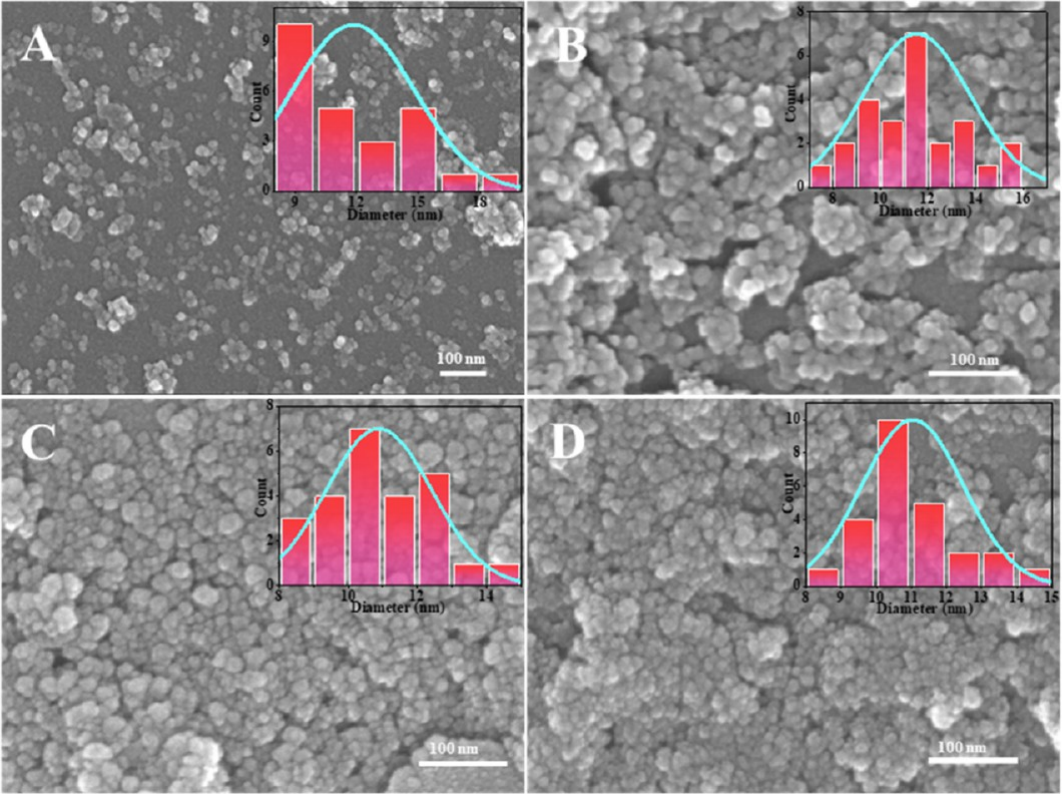
SEM images of silica prepared with different TEOS amounts: (A) 0.04; (B) 0.07; (C) 0.12; and (D) 0.15 mol/L.

**Figure 14:**
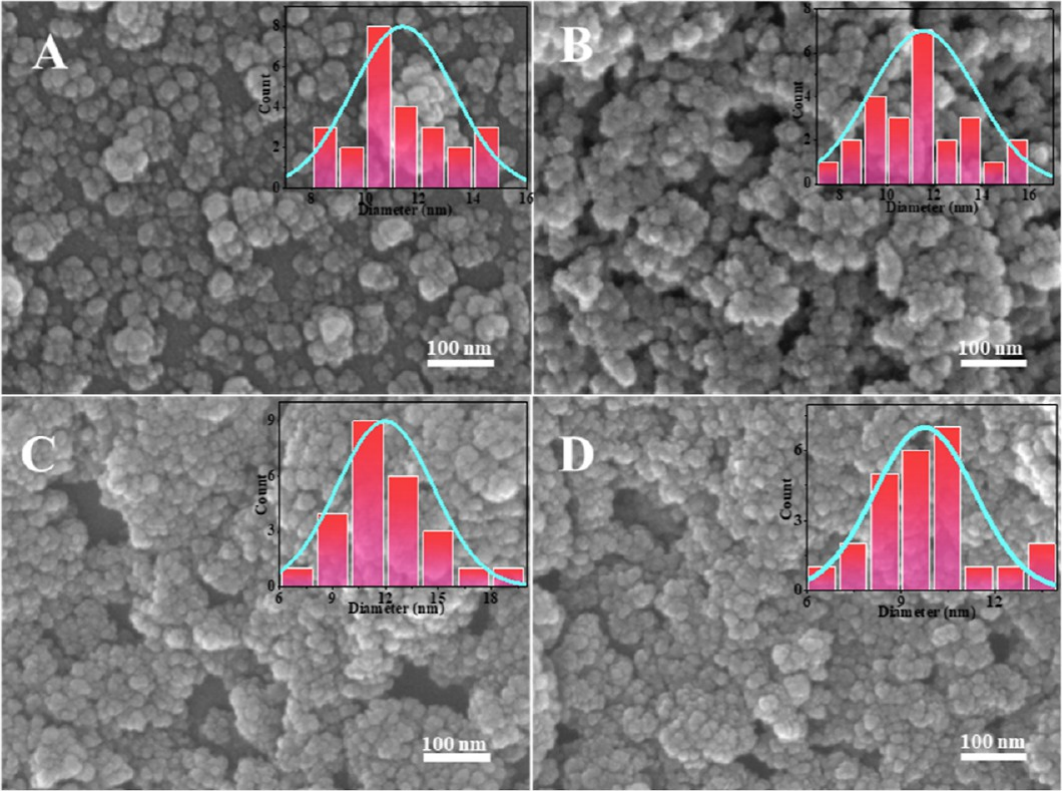
SEM images of silica prepared with different amounts of ammonia: (A) 0.02; (B) 0.04; (C) 0.06; and (D) 0.08 mol/L.

Figure 13A–D shows the dispersion of silica as a function of the amount of TEOS. Silicon dioxide from monodisperse to massive aggregation with decreasing the amount of TEOS from 0.04 to 0.15 mol/L. The reason for this is possible that the increase of TEOS causes the polymeric surfactant’s is long chains that cannot block TEOS well, which leads to the serious agglomeration of silica. It can be seen that no matter how the amount of ammonia–water is changed, the particle size or morphology of silica will not be changed (Figure 14). Note that a more dispersed ultrasmall silica can be obtained by adding a small amount of ammonia–water.

**Figure 15:**
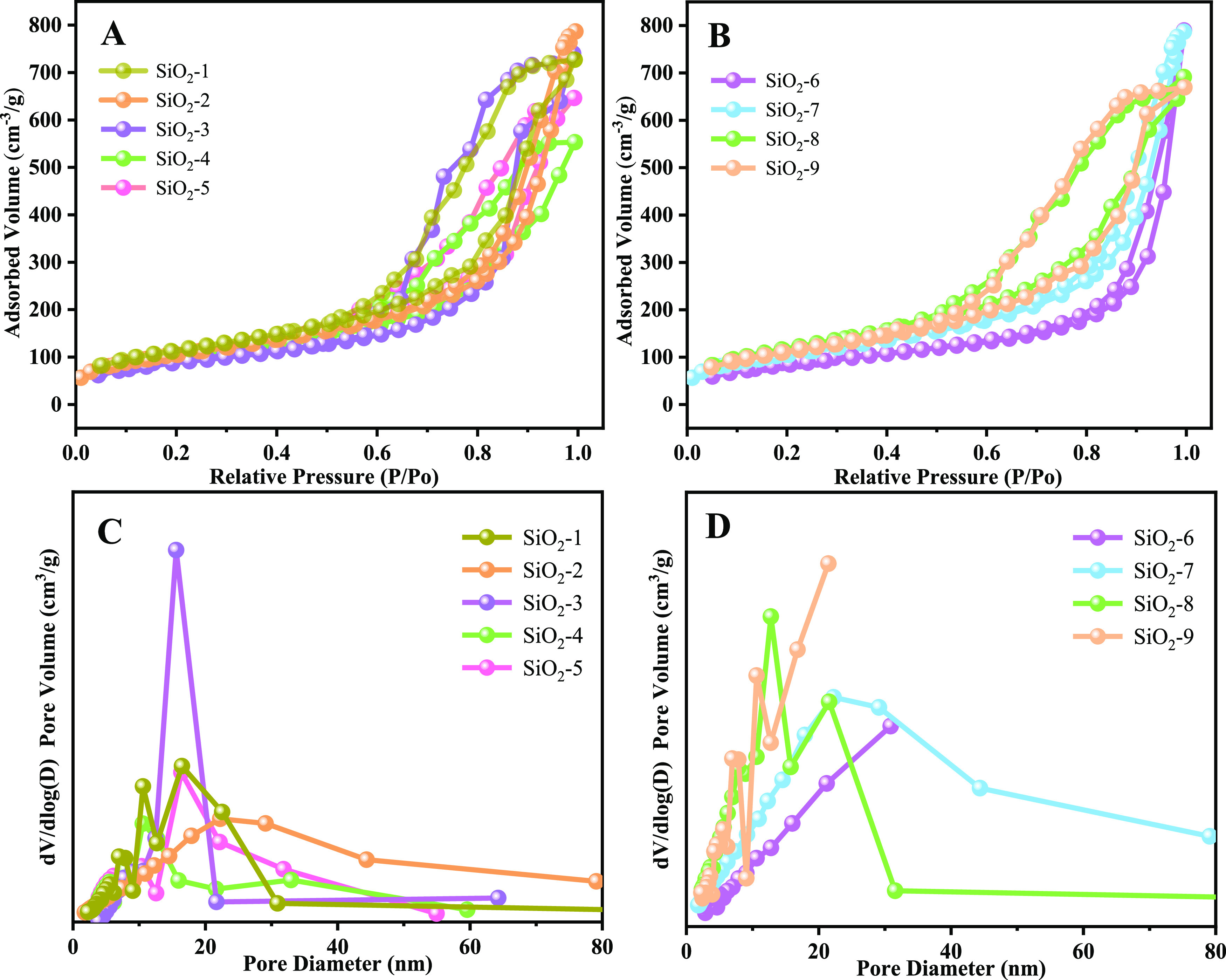
N_2_ adsorption–desorption isotherms of polymeric surfactants with different oil/water ratios (A), different TEOS additions (B), and pore size distributions (C and D).

**Figure 16:**
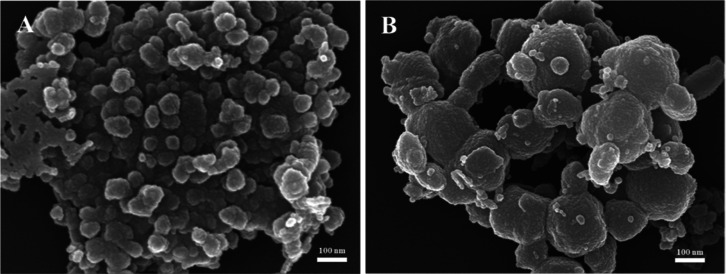
SEM images of silica prepared by CTAB at different magnifications: low magnification (A) and high magnification (B).

The specific surface area and pore size distribution of SiO_2_ prepared from PIBSA-DETA at a reaction ratio of 2.0 were investigated by using N_2_ adsorption–desorption experiments (Figure 15). As shown in Figure 15A,B, a strong hysteresis loop was observed for SiO_2_ in the high relative pressure region (0.6 < *P*/*P*_0_ < 1.0) with a typical type-II isotherm curve, indicating the existence of a mesoporous structure. The specific surface area of silica is 422.81, 384.02, 333.58, 358.22, and 373.55 m^2^/g in Figure 16A, and the corresponding pore sizes are 10.64, 12.67, 11.35, 8.01, and 9.85 nm, respectively (Table 2). These values are consistent with the previous SEM and TEM images (Figure 11 and Figure 12). In addition, these silicas are all mesoporous structures, as shown in Figure 15C. When the oil/water ratio decreases, the specific surface area first decreases and then increases. The reason for this trend is that as the oil decreases and the water increases, the oil–water interface becomes larger, making the aggregation of silica decrease, as shown in Figure 11A,B. When the oil/water ratio is 3:7, ultrasmall nanosilica gathers to form a dense sheet-like structure (Figure 11C), and do not completely form a sphere, so the specific surface area is the smallest. With the further reduction of the oil/water value, the oil–water interface becomes relatively small, and the close accumulation between the small spheres forms a sphere, making the specific surface area increase consistent with the results in Figure 11D–E.

**Table 2:** Physicochemical Properties of Ultrasmall Nanosilica Prepared under Different Octane/Water Molar Ratios

material	octane amount/mL	water amount/mL	octane/water molar ratio	*S*_BET_ (m^2^/g)	*V*_P_ (cm^3^/g)	*D* (nm)
SiO_2_-1	10	90	0.11	422.81	1.12	10.64
SiO_2_-2	20	80	0.25	384.02	1.21	12.67
SiO_2_-3	30	70	0.43	333.58	0.94	11.35
SiO_2_-4	40	60	0.67	358.22	0.71	8.01
SiO_2_-5	50	50	1.00	373.55	0.91	9.85

The specific surface areas of silica in Figure 15B are 324.91, 384.02, 436.59, and 409.45 m^2^/g, and the corresponding pore sizes are 15.03, 12.67, 9.79, and 10.11 nm (Table 3), respectively. Similarly, these silicas are all mesoporous structures, as shown in Figure 15D. Controlling the oil/water ratio, the specific surface areas show a trend of increasing and then decreasing with the increase of TEOS amount. When TEOS is in small amounts, silica can be uniformly dispersed on the oil–water interface, making the possibility of aggregation reduced and behaving in a dispersed state. With the increase of TEOS, the silica is increasing, the oil–water interface remains unchanged. The silica shows a state of tight accumulation and the existence of stacked pores, and the specific surface area increases. As TEOS is excessive, the tight accumulation of silica reduces the porosity, making the specific surface area decrease, and the above results are consistent with the results shown in Figure 13.

**Table 3:** Physicochemical Properties of Ultrasmall Nanosilica Prepared under Different TEOS Amounts

material	TEOS amount/mL	*S*_BET_ (m^2^/g)	*V*_P_ (cm^3^/g)	*D* (nm)
SiO_2_-6	3.6	324.91	1.22	15.03
SiO_2_-7	7.2	384.02	1.21	12.67
SiO_2_-8	10.8	436.59	1.06	9.79
SiO_2_-9	14.4	409.45	1.03	10.11

The FT-IR spectra of silica nanoparticles prepared by different reaction ratios of polymeric surfactant PIBSA-DETA demonstrated the functional groups (Figure S15). Figure S15A–C shows the intense and broad adsorption band appearing at 1112 cm^–1^ is assigned to asymmetric stretching vibrations of Si–O–Si,^41^ which suggests that a dense silica network was formed. In addition, one can observe the other important infrared vibrations of silica: the bending mode of Si–O–Si appearing at about 472 and 805 cm^–1^, and the stretching of the O–H appearing at 3430 cm^–1^. The FT-IR spectra indicate that the silica was successfully formed in the preparation. There is no obvious difference in the FT-IR spectra of silica particles. This suggests that the addition of small amounts of different reaction ratios of the polymeric surfactant PIBSA-DETA does not lead to significant change in the chemical composition of the silica nanoparticles.

XRD is one of the analyses used to determine the structural properties of nanoparticles. The XRD pattern for silica nanoparticles prepared by different reaction ratios of the polymeric surfactant PIBSA-DETA nanoparticles is shown in Figure S16. The XRD patterns showed that (Figure S16A–C) all have wide peaks at 23°, indicating that the synthesized silica is purely amorphous. TGA of silica before and after calcination is presented in Figure S17. The TGA curve of silica before calcination (Figure S17A) showed two weight loss steps occurring at 60–650 °C. The first weight loss from 60 to 100 °C with weight loss percentage of 0.8% belongs to the removal of adsorbed and bound water from SiO_2_, and between 250 and 650 °C showed a large mass loss of about 8 wt % of SiO_2_, which is thought to be caused by the decomposition of the polymeric surfactant and the oil phase. As shown in Figure S17B, the TG curve of calcined silica has 1 mass loss zone, and the weight loss of silica after calcination is 1.5% at 60–100 °C, which is the removal of adsorbed and bound water from the sample. The mass loss is not obvious when the temperature is >150 °C, which indicates that the sample has good thermal stability.

#### 3.2.3 Effect of Other Surfactants on the Preparation of Silica

Based on the above preparation of ultrasmall nanosilica, the surfactant CTAB was used to replace PIBSA-X, and the SEM images showed that the silica was heavily agglomerated together, forming irregularly shaped silica of 100–850 nm, with no small particles seen (Figure 16A,B). This is because the small molecular weight of CTAB, which is not a long-chain structure, cannot act as a barrier in the process of TEOS hydrolysis, so that a silica of about 10 nm cannot be obtained.

### 3.3 Formation Mechanism of Ultrasmall Nanosilica and Their Aggregates

**Figure 17:**
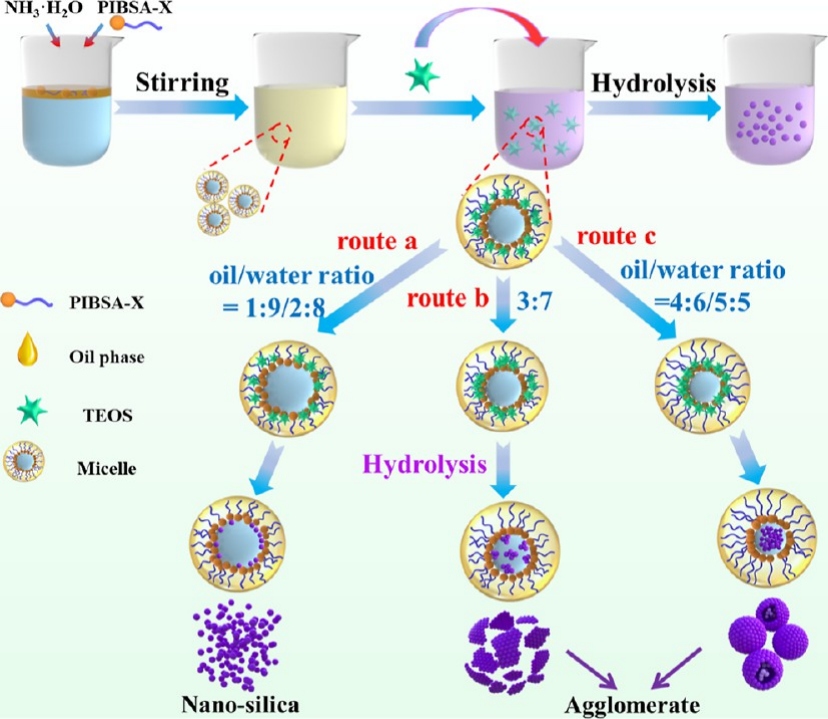
Schematic illustration of the formation mechanism of ultrasmall nanosilica and their aggregates by varying the oil/water ratio.

The procedure for preparing silica nanoparticles is illustrated in Figure 17. The above results clearly show that spherical silica materials with different morphologies can be successfully fabricated by using the emulsion method. The morphology and nanosphere dispersion can be controlled by tuning the ratio of the oil to water. We propose the mechanism shown in Figure 17 to explain the formation of silica of different morphologies. When the water phase (water and ammonia) was added to the oil phase (base oil and PIBSA-DETA), stable water-in-oil (W/O) type droplets formed. It has been shown that TEOS is hydrolyzed at the oil–water interface to form silica after adding TEOS. When the oil/water ratio is 1:9 or 2:8 (route a), the volume of the water phase is larger, and the silica obtained by hydrolysis will enter the water phase through the gap at the oil–water interface. The surfactant and silica are closely adsorbed together because of the hydration, preventing the agglomeration of silica during the collision. This is aligned with Figures 11A,B and 12A,B. However, when the oil/water ratio is 3:7 (route b), the collision frequency of silica in the aqueous phase is higher and the ultrasmall nanosilica aggregates, thereby forming lamellar silica (Figures 11C and 12C). When the oil/water ratio is further changed to 4:6 and 5:5 (route c), the ultrasmall silica is more likely to agglomerate into large silica particles (Figures 11D,E and 12D,E). The reason for the existence of large pore-breaking spheres is that the reduction of the aqueous phase leads to a lot of silica not entering the aqueous phase but still arranged at the oil–water interface, and the large pore-breaking spheres are obtained after the surfactant is removed by postprocessing (Figures 11E and 12E).

## 4 Conclusions

In summary, we prepared a range of inexpensive and environmentally friendly PIBSA-X polymeric surfactants, which were then used as the isolating agent in the preparation of nano silica, resulting in ultrasmall nanosilica spheres of around 10 nm in general. We have reported a simple approach to switch the morphology of silica nanospheres by controlling the oil/water ratio and the quantity of ammonia and TEOS added to the emulsion system. The synthesized solid silica nanoparticles showed good uniformity and size distribution. In this paper, we prepared PIBSA-polyenolamine polymer surfactant, PIBSA-polyol polymer surfactant, and PIBSA-polyol amine polymer surfactant. It was also found that PIBSA-polyenamine polymeric surfactants appeared in micrometer-sized vesicles after emulsification. Therefore, in the subsequent preparation of silica, all 10 nm silica could be obtained, which closed to commercial silica microspheres, confirming the universality of this series of polymeric surfactants for the preparation of ultrasmall nanosilica. Modulating the oil/water ratio, different morphologies of nanosilica aggregates evolve from ultrasmall nanosilica to nanosheets, and then to large microspheres, which also confirms the synthesis mechanism of ultrasmall nanosilica. This work provides a precise and simple synthetic method for the development of ultrasmall nanosilica and the low cost of raw materials, which has good potential to be industrialized.

## Supporting Information Available

Preparation of surfactants with different hydrophilic groups, 1H NMR analysis of PIBSA and PIBSA-DETA, FT-IR analysis of polymeric surfactants, polarized micrographs and photomicrographs of emulsions with different reaction ratios of PIBSA-X and the FT-IR, and XRD and TG analysis of ultrasmall nanosilica (PDF)
