# Supplementary material for: Polymeric Surfactant (PIBSA-X) Facilitates the Formation of a Water-in-Oil Emulsion Reactor for the Preparation of Ultrasmall Nanosilica
Source: ACS Omega. 2023 Nov 14;8(47):44647–58. doi: 10.1021/acsomega.3c05335 (PMC10688208; doi:10.1021/acsomega.3c05335)
Supplement: Supplementary file 1 — ao3c05335_si_001.pdf [file ao3c05335_si_001.pdf]

Supporting information

Polymeric surfactant (PIBSA-X) facilitates the formation of water-in-oil emulsion reactor for preparation of ultra-small nano-silica

Rui Cao[a], Chun Wang[a], Chengliang Zhou[a], Yong Liu[b], Yating Yin[b], Haibao Chen[b], Feng Li[a], Wending Zhou[a], Meisong Xu[a], Wanliang Yang[a,c]*

[a] *School of Chemistry and Chemical Engineering, Guizhou University, Guiyang, 550025, China*

[b] *Guizhou Juneng Chemical Co., Ltd. Huishui County of Guizhou Province, 550601, PR China*

[c] *Guizhou Provincial Double Carbon and Renewable Energy Technology Innovation Research Institute, Guizhou University, Guiyang, 550025, China*

To whom correspondence should be addressed:

*TEL: +86-15985159596

E-mail: yangwanlianghhhh@163.com (W-L. Yang)

□ *Corresponding authors.*

Table of Contents

Part S1: Preparation method of materials.

SI01. Emulsification equipment.

Part S2: Characterisation

SI02. ^{1}H-NMR analysis of samples.

SI03. FT-IR spectra of PIBSA-diethylene glycol polymeric surfactant.

SI04. Emulsification stability analysis of PIBSA-diethylene glycol polymeric surfactant.

SI05. FT-IR spectra of PIBSA-EG polymeric surfactant.

SI06. Emulsification stability analysis of PIBSA-EG polymeric surfactant.

SI07. FT-IR spectra of PIBSA-TETA polymeric surfactant.

SI08. Emulsification stability analysis of PIBSA-TETA polymeric surfactant.

SI09. FT-IR spectra of PIBSA-urea polymeric surfactant.

SI10. Emulsification stability analysis of PIBSA-urea polymeric surfactant.

SI11. FT-IR spectra of PIBSA-(2-Methylaminoethanol) polymeric surfactant.

SI12. Emulsification stability analysis of PIBSA-(2-Methylaminoethanol) polymeric surfactant.

SI13. FT-IR spectra of PIBSA-TEA polymeric surfactant.

SI14. Emulsification stability analysis of PIBSA-TEA polymeric surfactant.

SI15. FT-IR analysis of ultra-small nano-silica.

SI16. XRD analysis of ultra-small nano-silica.

SI17. TG analysis of ultra small nano-silica.

Part I: Preparation method of materials

Preparation of surfactants with different hydrophilic groups:

(1) Preparation of polymeric surfactants with different reaction ratios

Under solvent-free conditions, a certain amount of polyisobutylene succinic anhydride (PIBSA) was added to a three-necked flask, heated to about 90 °C, stirred, PIBSA is fluid, and a certain ratio (PIBSA:X=0.5, 1.0, 2.0) of different hydrophilic groups of raw materials (triethylenetetetramine, urea, ethylene glycol, diethylene glycol, triethylene glycol, N,N-dimethylethanol. The reaction was carried out under a nitrogen atmosphere to prevent spillage. Under a nitrogen-protected atmosphere, the temperature was gradually increased to 160°C and the reaction was kept at a constant temperature for 6 h.

(2) Preparation of polymeric surfactants with different reaction times

Under solvent-free conditions, a quantity of polyisobutylene succinic anhydride (PIBSA) was added to a three-necked flask, heat the temperature to about 90 °C, stir, PIBSA is fluid, slowly add 2.0 proportions of different hydrophilic groups of raw materials to prevent overflow When the mixture is homogeneous, nitrogen is introduced to remove the air from the reactor. The temperature is gradually increased to 160 °C by nitrogen protection and the reaction is kept at a constant temperature for a certain time (4 h, 5 h, 6 h, 7 h, 8 h).

(3) Preparation of polymeric surfactants at different reaction temperatures

Under solvent-free conditions, a quantity of polyisobutylene succinic anhydride (PIBSA) was added to a three-necked flask, heat the temperature to about 90 °C, stir, PIBSA is fluid, slowly add 2.0 ratio of different hydrophilic groups of raw materials (triethylenetetetramine, urea, ethylene glycol, diethylene glycol, triethylene glycol, N, N-dimethylethanol, 2-methylaminoethanol, triethanolamine) to prevent overflow. When the mixture is homogeneous, pass nitrogen to remove air from the reactor. Increase to the specified temperatures (T=140 °C, 145 °C, 150 °C, 155 °C, 160 °C, 165 °C), allow to stabilise and keep the reaction at a constant temperature for 6 h.

As shown in the text silica preparation method.

SI01. Emulsification equipment

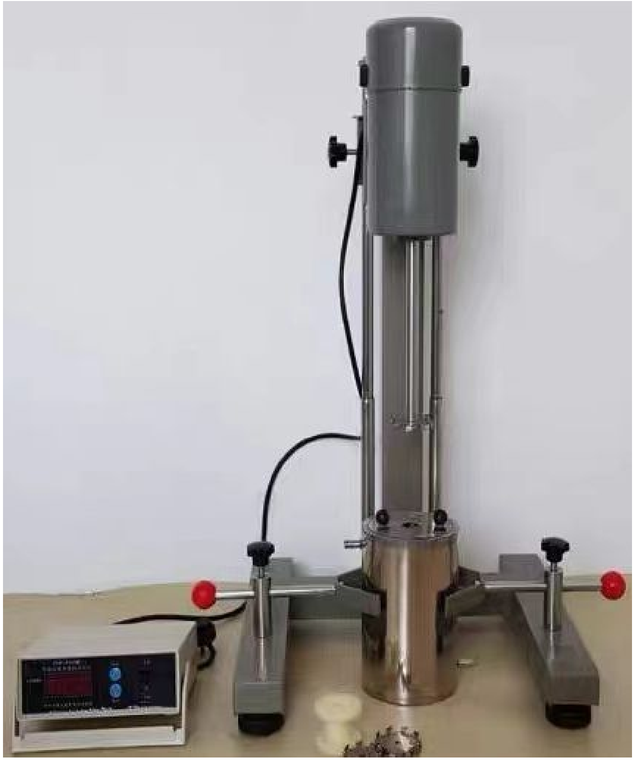

Figure S1. Emulsification equipment

Part S2: Characterisation

SI02. ^{1}H-NMR analysis of samples

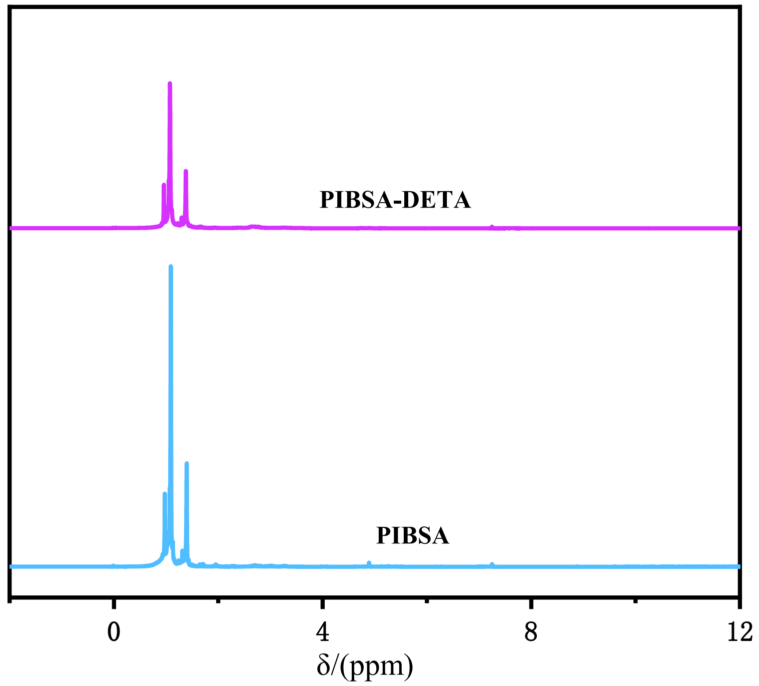

Figure S2. ^{1}H-NMR of PIBSA and PIBSA-DETA polymer surfactant.

(Ⅱ) FT-IR analysis of polymeric surfactants and emulsion stability analysis

SI03. FT-IR spectra of PIBSA-diethylene glycol polymeric surfactant

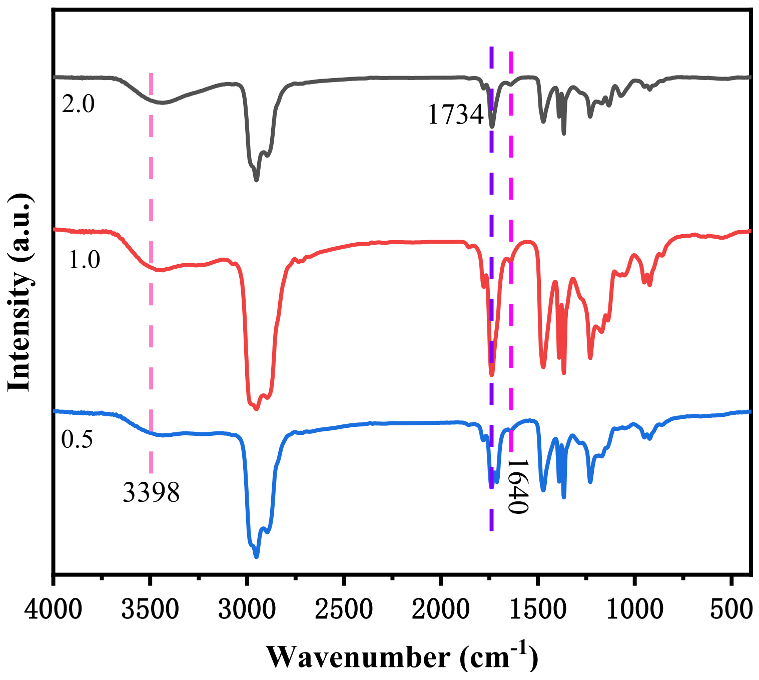

Figure S3. FT-IR spectra of PIBSA-DEG polymeric surfactant with different reaction ratios (PIBSA: DEG=0.5, 1.0, 2.0).

SI04. Emulsification stability analysis of PIBSA-diethylene glycol polymeric surfactant.

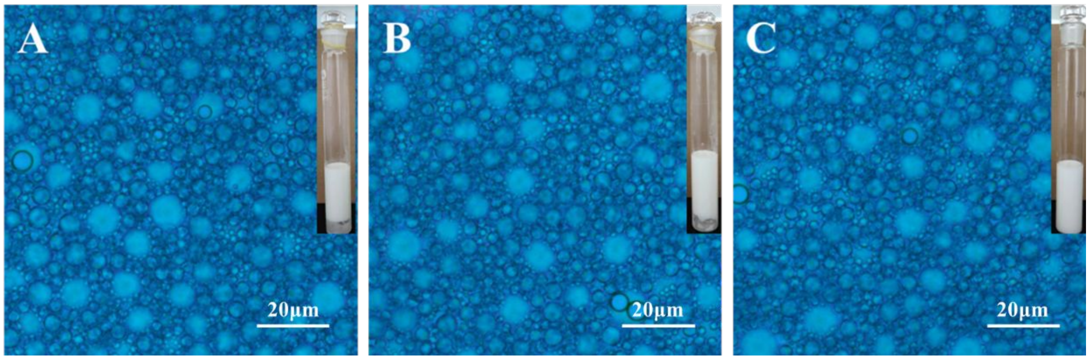

Figure S4. Polarized micrographs and photomicrographs of emulsions with different reaction ratios of PIBSA-DEG polymer surfactants: A 0.5, B 1.0, C 2.0.

SI05. FT-IR spectra of PIBSA-EG polymeric surfactant.

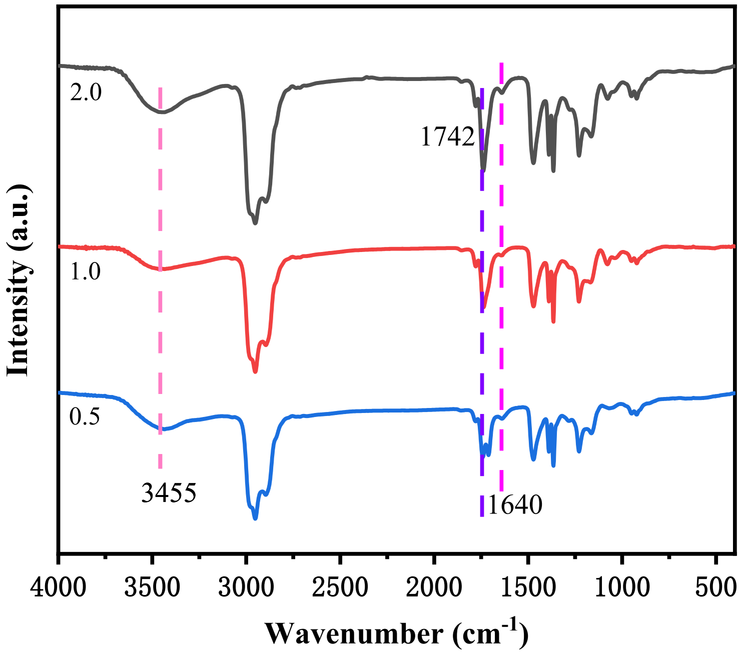

Figure S5. FT-IR spectra of PIBSA-EG polymeric surfactant with different reaction ratios (PIBSA: EG=0.5, 1.0, 2.0).

SI06. Emulsification stability analysis of PIBSA-EG polymeric surfactant.

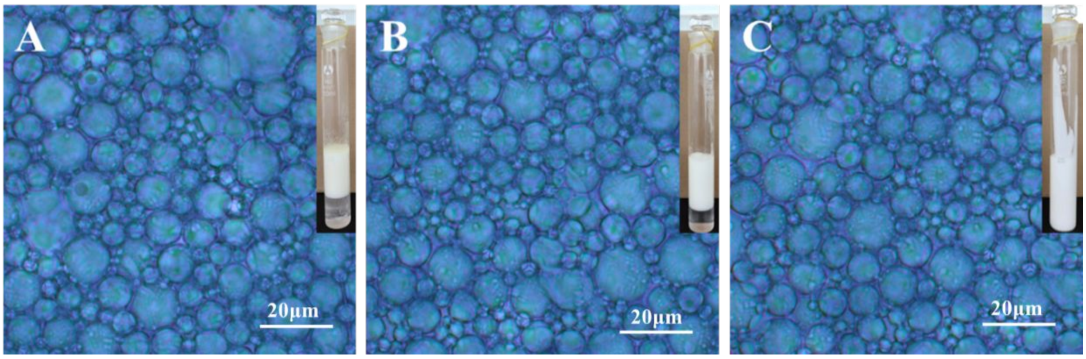

Figure S6. Polarized micrographs and photomicrographs of emulsions emulsified with different reaction ratios of PIBSA-EG polymer surfactants: A 0.5, B 1.0, C 2.0.

SI07. FT-IR spectra of PIBSA-TETA polymeric surfactant

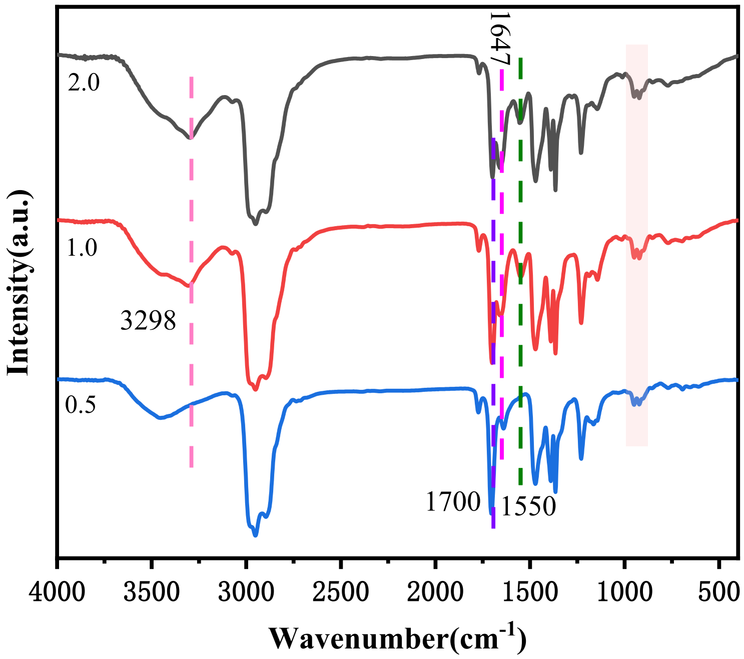

Figure S7. FT-IR spectra of PIBSA- TETA polymeric surfactant with different reaction ratios (PIBSA: TETA =0.5, 1.0, 2.0).

SI08. Emulsification stability analysis of PIBSA-TETA polymeric surfactant

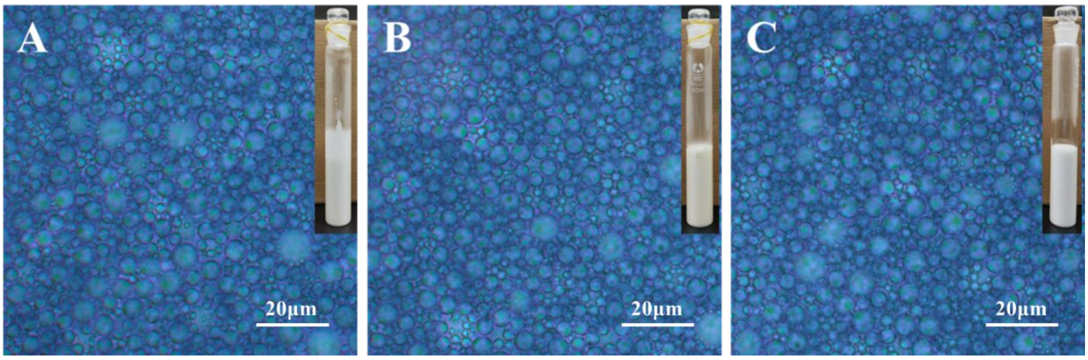

Figure S8. Polarized micrographs and photomicrographs of emulsions emulsified with different reaction ratios of PIBSA- TETA polymer surfactants: A 0.5, B 1.0, C 2.0.

SI09. FT-IR spectra of PIBSA-urea polymeric surfactant.

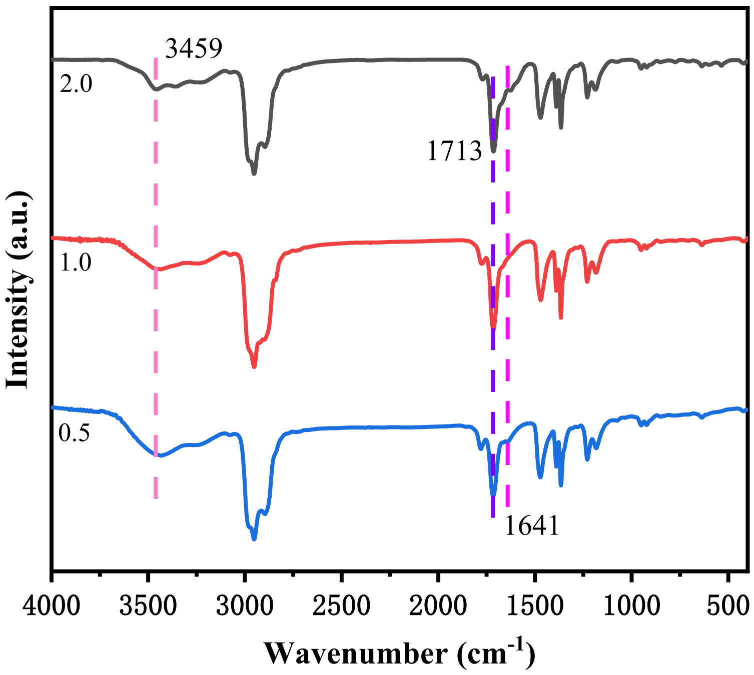

Figure S9. FT-IR spectra of PIBSA- Urea polymeric surfactant with different reaction ratios (PIBSA: Urea =0.5, 1.0, 2.0).

SI10. Emulsification stability analysis of PIBSA-urea polymeric surfactant.

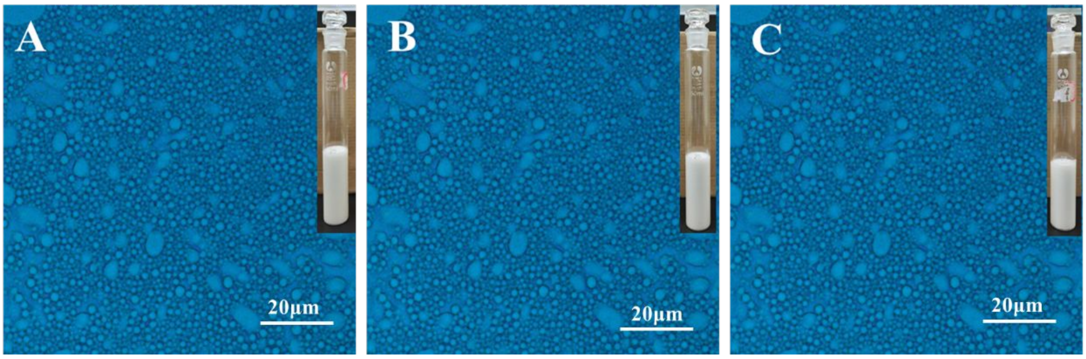

Figure S10. Polarized micrographs and photomicrographs of emulsions emulsified with different reaction ratios of PIBSA- Urea polymer surfactants: A 0.5, B 1.0, C 2.0.

SI11. FT-IR spectra of PIBSA-(2-Methylaminoethanol) polymeric surfactant.

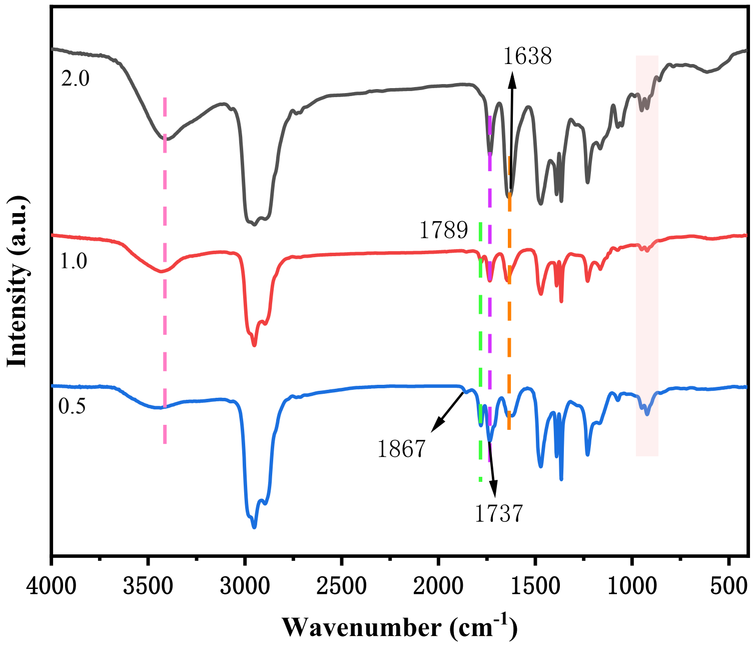

Figure S11. FT-IR spectra of PIBSA- (2-Methylaminoethanol) polymeric surfactant with different reaction ratios (PIBSA: (2-Methylaminoethanol) =0.5, 1.0, 2.0).

SI12. Emulsification stability analysis of PIBSA-(2-Methylaminoethanol) polymeric surfactant.

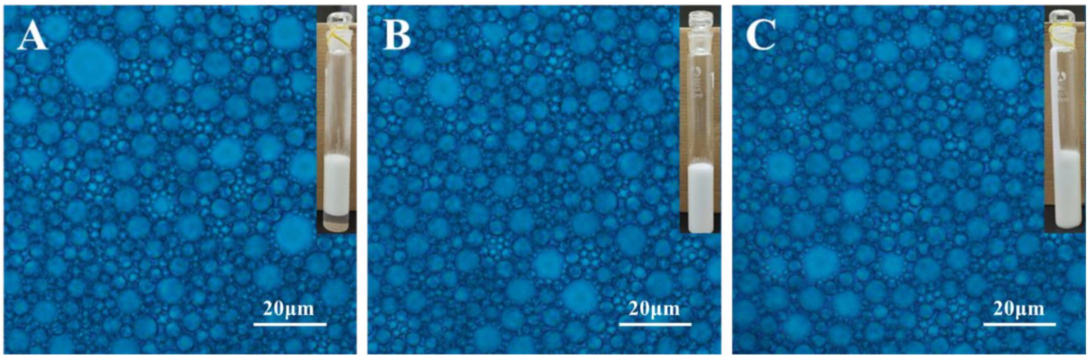

Figure S12. Polarized micrographs and photomicrographs of emulsions emulsified with different reaction ratios of PIBSA-(2-Methylaminoethanol) polymer surfactants: A 0.5, B 1.0, C 2.0.

SI13. FT-IR spectra of PIBSA-TEA polymeric surfactant.

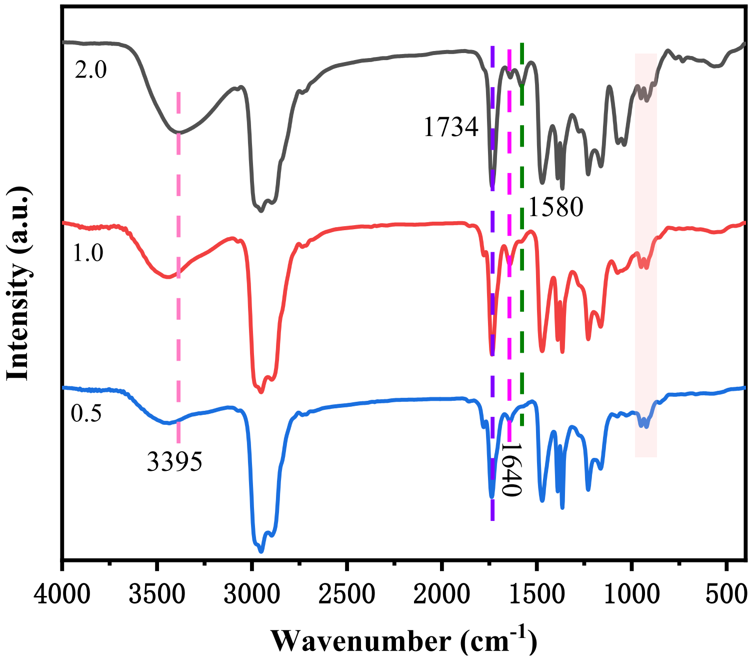

Figure S13. FT-IR spectra of PIBSA- TEA polymeric surfactant with different reaction ratios (PIBSA: TEA =0.5, 1.0, 2.0).

SI14. Emulsification stability analysis of PIBSA-TEA polymeric surfactant.

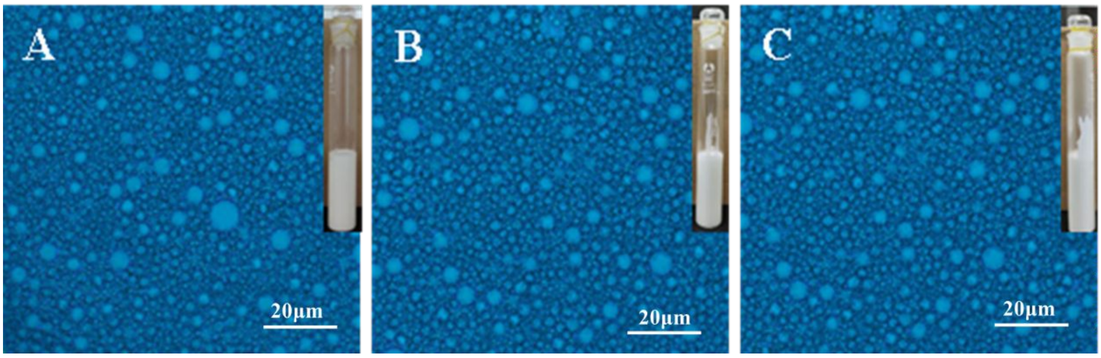

Figure S14. Polarized micrographs and photomicrographs of emulsions emulsified with different reaction ratios of PIBSA- TEA polymer surfactants: A 0.5, B 1.0, C 2.0.

SI15. FT-IR analysis of ultra-small nano-silica.

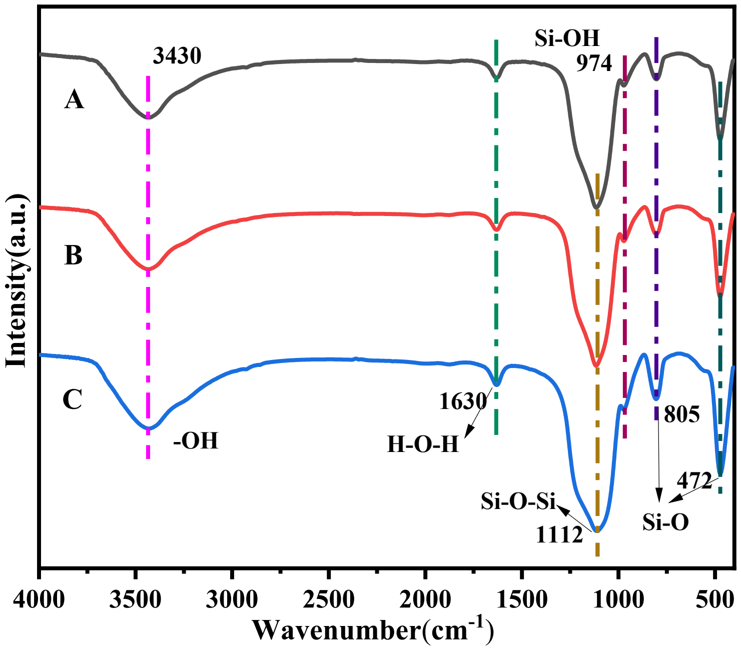

Figure S15. The FT-IR spectra of the silica nanoparticles synthesized with different reaction ratios of PIBSA-DETA (A) reaction ratio of 2.0; (B) reaction ratio of 1.0 and (C) reaction ratio of 0.5.

SI16. XRD analysis of ultra-small nano-silica.

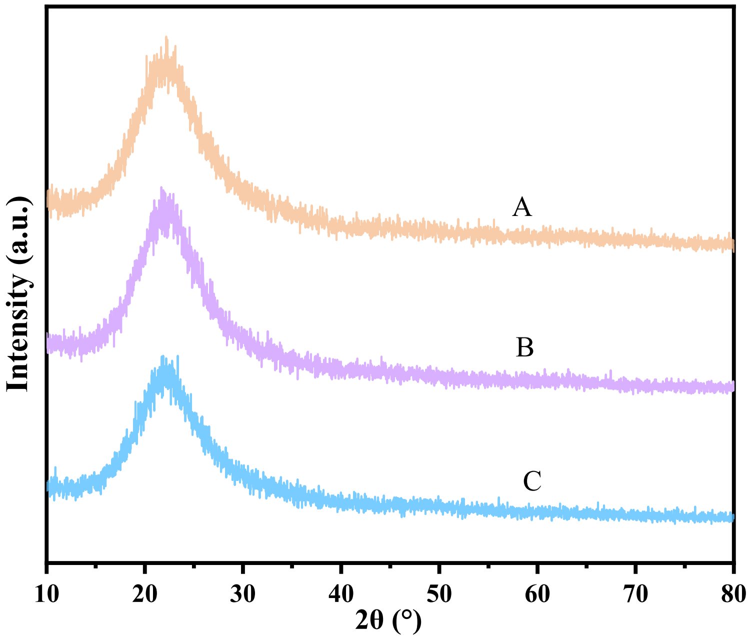

Figure S16. XRD of ultra-small silica nanoparticles, with surfactants PIBSA-DETA (2.0, A), PIBSA-DETA (1.0, B), PIBSA-DETA (0.5, C).

SI17. TG analysis of ultra small nano-silica.

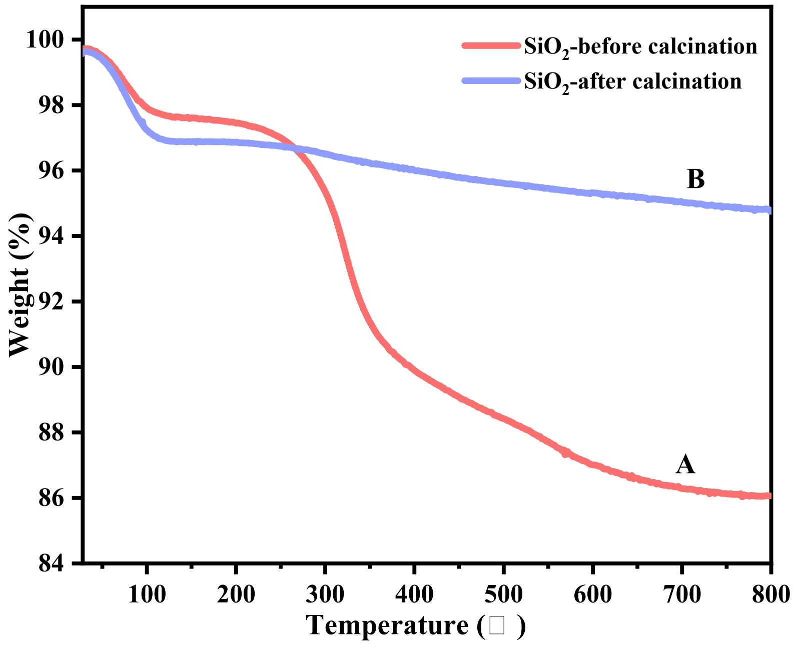

Figure S17. TG of ultra-small nano-silica before and after calcination.
